# Mesenchymal stem cells rejuvenate cardiac muscle after ischemic injury

**DOI:** 10.18632/aging.101718

**Published:** 2019-01-06

**Authors:** Busheng Zhang, Jing Zhang, Dan Zhu, Ye Kong

**Affiliations:** 1 Department of Cardiac Surgery, Shanghai Chest Hospital, Shanghai Jiao Tong University, Shanghai 200030, China; * Equal contribution

**Keywords:** mesenchymal stem cells (MSCs), myocardial infarction (MI), CD146, transplantation

## Abstract

Previous studies have shown that transplantation of mesenchymal stem cells (MSCs) enhances myocardial regeneration after myocardial infarction (MI), primarily resulting from the production and release of trophic growth factors and cytokines by MSCs. However, effects of MSCs or a subtype of MSCs on the ageing of injured cardiac muscle cells (CMCs) are limitedly known. Here, we addressed this question. CD146+ MSCs were isolated from total MSCs (tMSCs), and their effects on injured CMCs were assessed. *In vivo*, transplantation of isogenic CD146+ MSCs into MI-mice increased the proliferation of CMCs and reduced apoptosis of CMCs in a significantly higher degree than transplantation of tMSCs, resulting in significant improvement of the heart function. *In vitro*, CMCs were co-cultured under hypoxia condition with CD146+MSCs or tMSCs. We found that CD146+MSCs increased the proliferation of CMCs and reduced apoptosis of CMCs in a significantly higher degree, compared to tMSCs, likely resulting from reduction of aging-associated cellular reactive oxygen species in CMCs. Together, these data suggest that MSCs rejuvenate CMCs after ischemic injury and a subtype of MSCs, CD146+ MSCs, appears to have higher potential in coordinating this process.

## Introduction

Coronary heart disease (CHD) is a prevalent disease with high morbidity. Prolonged impairment of physiological supply from coronary blood vessels results in ischemic cardiomyopathy and even myocardial infarction (MI), which causes heart failure [1]. Prevention from the dysfunction and loss of myocardial cells, and augmentation of the regeneration of cardiomyocytes after MI are both critical approaches leading to a successful CHD therapy [2].

Previous studies have demonstrated the promise of using bone marrow derived cells in promoting the recovery and regeneration of the infarcted myocardium, while effective cells among bone marrow derived cells appeared to mesenchymal stem cells (MSCs) [3–6]. Since transplantation of MSCs in the infarcted heart tissue led to a signiﬁcant decrease in scar area without improvement of regional myocardial contractility, differentiation of MSCs into myocytes to couple with existing myocytes unlikely accounted for the reduction in scar area [7]. On the other hand, more and more studies support that MSCs improve the post-MI myocardial primarily from production and release of trophic growth factors and cytokines by MSCs to affect the insulted myocardial cells [8]. Also, previous evidence that the protective effects of MSCs on injured myocardial cells may also include anti-aging effects [9–11].

MSCs are characterized by expression of specific surface markers, Sca-1, CD105 and CD90, and by lack of expression for CD45, CD34 and HLA-DR [12–14]. In addition, MSCs maintain multipotent differential potential to be induced differentiation into adipocytes, osteocytes and chondrocytes, respectively [15]. However, MSCs are not a group of identical cells [16–18]. Some genes were differentially expressed in MSCs, suggesting presence of MSC subtypes that contribute to different cell lineage in future [19]. CD146 in a marker that expresses in capillary pericytes [20]. Since the anti-fibrotic effects of MSCs are associated with their regulation of activities of matrix metalloproteinases (MMPs), which break down collagens as well as tissue inhibitors of metalloproteinases, and with the immunosuppressive function of MSCs, pericyte-prone MSCs with such functionality may play crucial roles in the post-MI regeneration of myocardial cells [21]. Thus, we hypothesize that MSCs may have a subpopulation that expresses CD146, and this subpopulation (CD146+MSCs) may play a more pronounced role compared to total MSCs (tMSCs). This hypothesis was thus assessed in the current study.

CD146+ MSCs were isolated from total MSCs (tMSCs), and their effects on injured CMCs were assessed. *In vivo*, transplantation of isogenic CD146+ MSCs into MI-mice increased the proliferation of CMCs and reduced apoptosis of CMCs in a significantly higher degree than transplantation of tMSCs, resulting in significant improvement of the heart function. *In vitro*, CMCs were co-cultured under hypoxia condition with CD146+MSCs or tMSCs. We found that CD146+MSCs increased the proliferation of CMCs and reduced apoptosis of CMCs in a significantly higher degree, compared to tMSCs, likely resulting from reduction of aging-associated cellular reactive oxygen species in CMCs.

## RESULTS

### CD146+MSCs represent a small population of tMSCs

**Figure 1:**
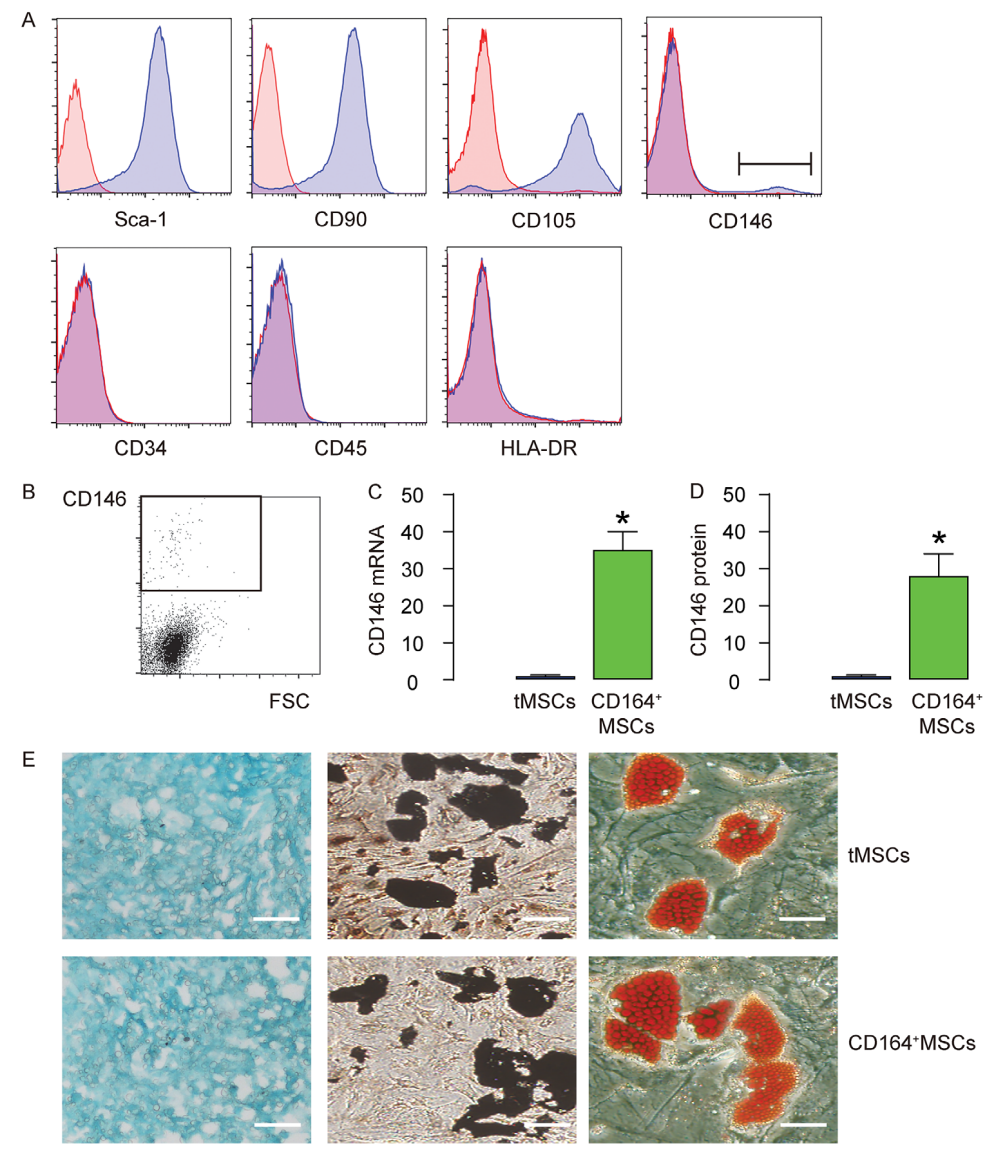
**CD146+MSCs represent a small population of total tMSCs.** (**A**) The surface markers for mouse MSCs (Sca-1, CD105, CD90 CD45, CD34, HLA-DR and CD146) were examined. (**B**) FAC sorting of CD146+ cells from tMSCs. (**C-D**) RT-qPCR (**C**) and ELISA (**D**) for CD146 in CD146+MSCs and tMSCs. (**E**) Differentiation assay for tMSCs and CD146+MSCs into chondrocytes followed by alcian blue staining (left), into osteocytes followed by Von kossa staining (middle), and into adipocytes followed by Oil red O staining (right). *p<0.05. N=5. Scale bars are 100 µm.

We isolated MSCs from mice. The surface markers for mouse MSCs were examined. We found that the isolated MSCs were all expressed high Sca-1, CD105 and CD90, but expressed null CD45, CD34 and HLA-DR, consistent with the well-established mouse MSC phenotype (Figure 1A). In addition, we examined the CD146 expression in MSCs and found a very small population that was positive for CD146 (less than 3%, Figure 1A). This small population of CD146+MSCs was thus purified by flow cytometry (Figure 1B). The RT-qPCR and ELISA for CD146 were performed on CD146+MSCs versus tMSCs, showing about 35 times’ enrichment in mRNA (Figure 1C), and about 30 times’ enrichment in mRNA (Figure 1D). Next, we assessed the differentiation potential of CD146+MSCs versus tMSCs. We found that both were able to differentiate into chondrocytes, osteocytes or adipocytes in the corresponding differentiation induction media (Figure 1E). Thus, CD146+MSCs represent a small population of MSCs and can be isolated from tMSCs.

### CD146+MSCs have a better protective effect on heart function than tMSCs in MI-mice

**Figure 2:**
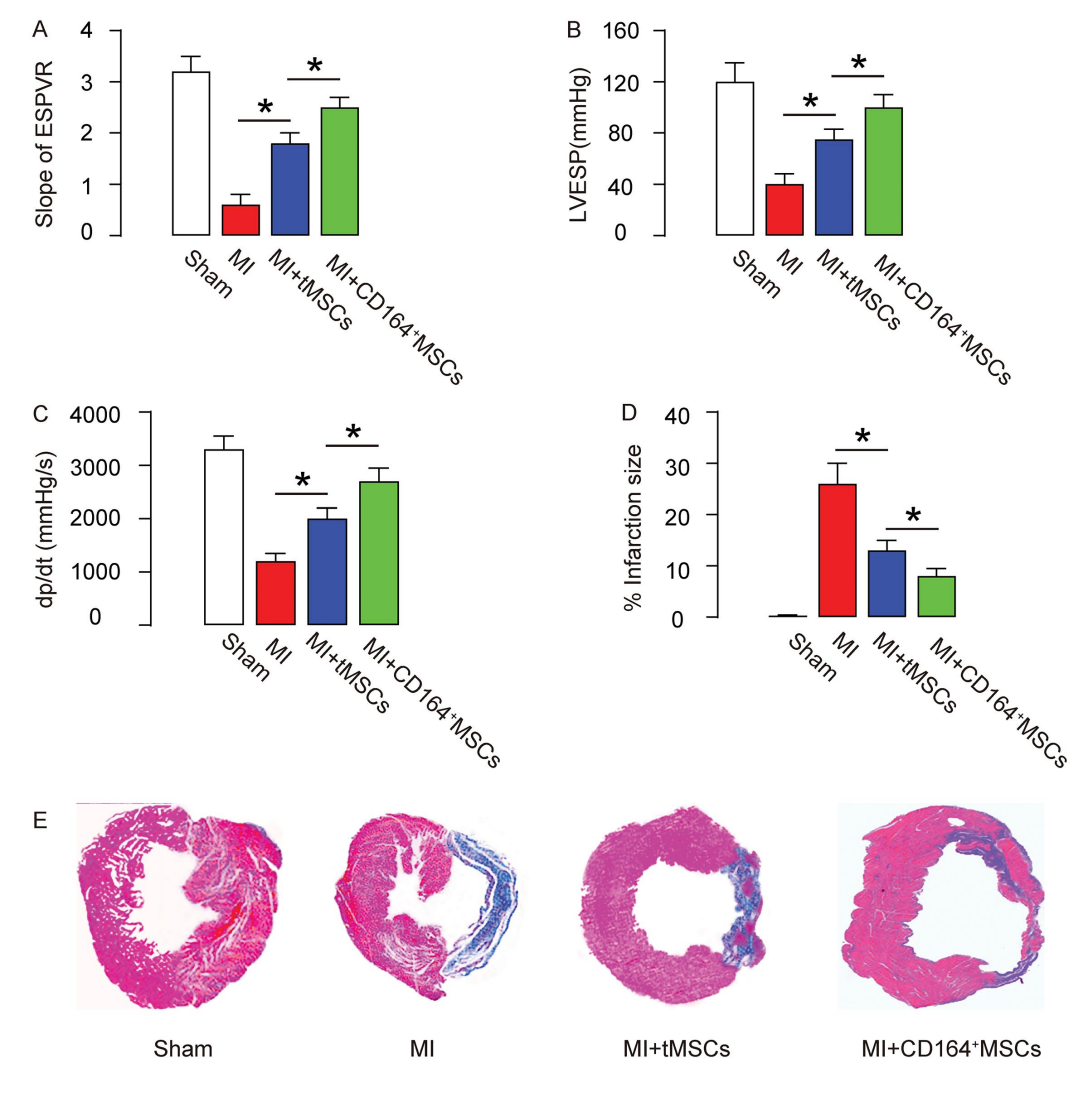
**CD146+MSCs have a better protective effect on heart function than tMSCs in MI-mice.** Four groups of mice were applied. Group 1, mice received sham surgery and injection of saline (Sham). Group 2, mice received MI surgery and injection of saline (MI). Group 3, mice received MI surgery and injection of tMSCs (MI+tMSCs). Group 4, mice received MI surgery and injection of CD164+MSCs (MI+CD164+MSCs). Four weeks later, the mouse heart function was assessed using ventricular catheterization. (**A**) End systolic pressure-volume relationship (ESPVR) (**B**) left ventricular end systolic pressure (LVESP) (**C**) Positive maximal pressure derivative (+dP/dt). (**D-E**) Masson's trichrome staining to determine the fibrotic heart tissue, shown by quantification (**D**), and by gross images (**E**). *p<0.05. N=10.

*In vivo*, the protective effects on heart function on MI-mice by CD146+MSCs were compared to tMSCs. Four groups of mice were applied. Group 1, mice received sham surgery and injection of saline (Sham). Group 2, mice received MI surgery and injection of saline (MI). Group 3, mice received MI surgery and injection of tMSCs (MI+tMSCs). Group 4, mice received MI surgery and injection of CD164+MSCs (MI+CD164+MSCs). Four weeks later, the mouse heart function was assessed using ventricular catheterization. We found that end systolic pressure-volume relationship (ESPVR) was significantly impaired in MI, compared to Sham. Transplantation of tMSCs significantly improved it, while transplantation of CD164+MSCs further significantly improved it, compared to transplantation with tMSCs (Figure 2A). Moreover, left ventricular end systolic pressure (LVESP) was significantly impaired in MI, compared to Sham. Transplantation of tMSCs significantly improved it, while transplantation of CD164+MSCs further significantly improved it, compared to transplantation with tMSCs (Figure 2B). Furthermore, positive maximal pressure derivative (+dP/dt) was significantly impaired in MI, compared to Sham. Transplantation of tMSCs significantly improved it, while transplantation of CD164+MSCs further significantly improved it, compared to transplantation with tMSCs (Figure 2C). Next, we used Masson's trichrome staining to assess the fibrosis levels after MI. We found that the infraction size after MI was significantly reduced by transplantation with tMSCs, shown by quantification (Figure 2D), and by the gross images (Figure 4E). The infraction size after MI was reduced more pronouncedly by transplantation with CD164+MSCs, compared to transplantation with tMSCs (Figure 2D-E). Thus, CD146+MSCs have a better protective effect on heart function than tMSCs in MI-mice.

### Higher CMC proliferation and less apoptosis are detected in MI-mice that receive transplantation of CD146+MSCs than those that receive tMSCs

**Figure 3:**
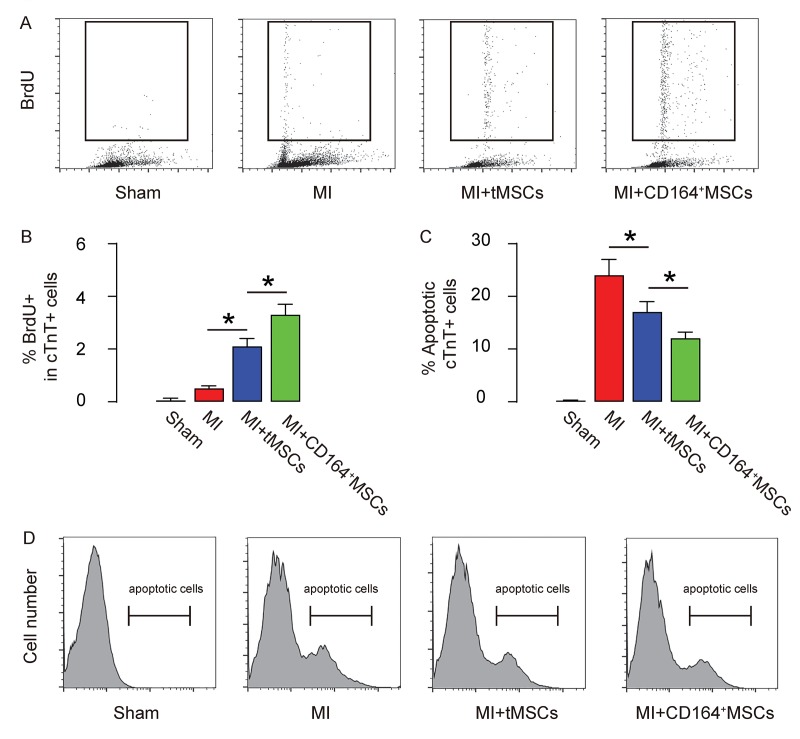
**Higher CMC proliferation and less apoptosis are detected in MI-mice that receive transplantation of CD146+MSCs than those that receive tMSCs.** CMCs were purified by flow cytometry based on their expression of Troponin T (cTnT). (A-B) BrdU+ CMCs were analyzed, shown by representative flow charts (**A**), and by quantification (**B**). (**C-D**) Apoptotic CMCs were analyzed in an Annexin V assay, shown by quantification (**C**), and by representative flow charts (**D**). *p<0.05. N=10.

The proliferation and apoptosis of CMC were analyzed to figure out the mechanism underlying improved protective effects on heart function by CD146+MSCs than by tMSCs. CMCs were purified by flow cytometry based on their expression of Troponin T (cTnT). BrdU+ cells or apoptotic cells were further analyzed in the cTnT+ populations. We found that transplantation of CD146+MSCs into MI-mice increased the proliferation of CMCs in a significantly higher grade than transplantation of tMSCs, shown by representative flow charts (Figure 3A), and by quantification (Figure 3B). Moreover, transplantation of CD146+MSCs into MI-mice s reduced apoptosis of CMCs in a significantly higher grade than transplantation of tMSCs, shown by quantification (Figure 3C), and by representative flow charts (Figure 3D). Thus, CD146+MSCs have a better protective effect on heart function than tMSCs, likely through increasing CMC proliferation and reducing CMC apoptosis.

### Enhanced cell proliferation by CD146+MSCs against hypoxia in cardiac muscle cells

**Figure 4:**
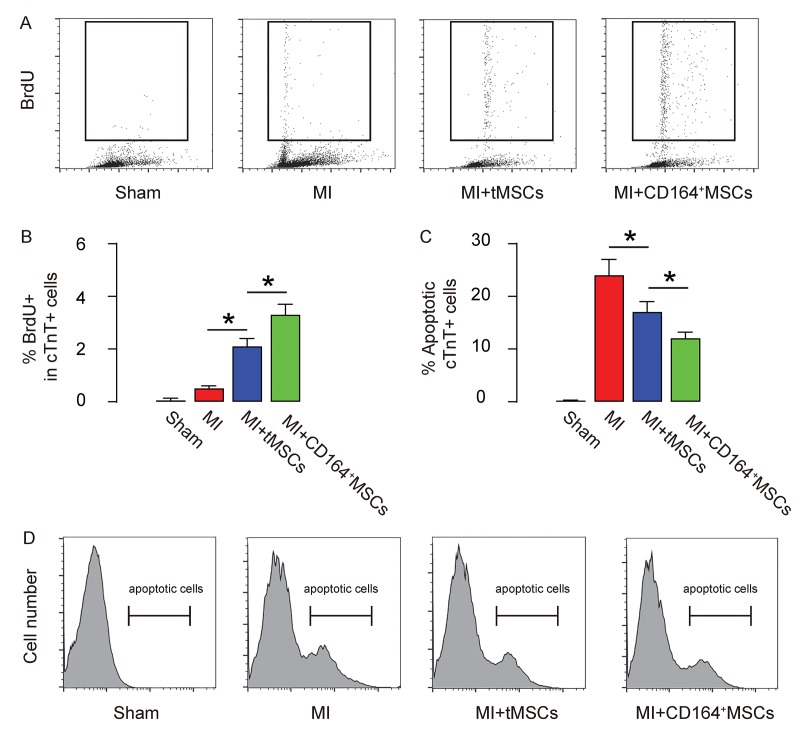
**Enhanced cell proliferation by CD146+MSCs against hypoxia in cardiac muscle cells.** (**A**) A transwell system was used to co-culture primary CMCs with different MSCs under hypoxia condition for 24 hours. Four conditions were applied. CMCs were plated at the lower plate. Condition 1, upper plate contained control media in normoxia condition (CTL). Condition 2, upper plate contained control media in hypoxia condition (Hypoxia). Condition 3, the upper plate contained same number of tMSCs (to the number of CMCs in lower plate) in hypoxia condition (Hypoxia+tMSCs). Condition 4, the upper plate contained same number of CD164+MSCs (to the number of CMCs in lower plate) in hypoxia condition (Hypoxia+CD164+MSCs). (B-C) BrdU+ CMCs were analyzed, shown by representative flow charts (B), and by quantification (C). *p<0.05. N=5.

In order to examine the protective effects of CD146+MSCs versus tMSCs on heart function of MI-mice, we used a transwell system to co-culture primary CMCs with different MSCs under hypoxia condition for 24 hours. Four conditions were applied. CMCs were plated at the lower plate. Condition 1, upper plate contained control media in normoxia condition (CTL). Condition 2, upper plate contained control media in hypoxia condition (Hypoxia). Condition 3, the upper plate contained same number of tMSCs (to the number of CMCs in lower plate) in hypoxia condition (Hypoxia+tMSCs). Condition 4, the upper plate contained same number of CD164+MSCs (to the number of CMCs in lower plate) in hypoxia condition (Hypoxia+CD164+MSCs) (Figure 4A). First, CMC proliferation was assessed by BrdU assay, showing that cell proliferation was slightly but significantly increased in hypoxia, compared to normoxia. Co-culture with tMSCs significantly improved the CMC proliferation compared to Hypoxia-only condition, while co-culture with CD164+MSCs further significantly increased the CMC proliferation compared to Hypoxia+tMSCs (Figure 4B-C). Thus, the CD146+MSCs induce more cell proliferation in CMCs than tMSCs under a hypoxia condition.

### Reduced cell apoptosis and ROS formation by CD146+MSCs against hypoxia in cardiac muscle cells

**Figure 5:**
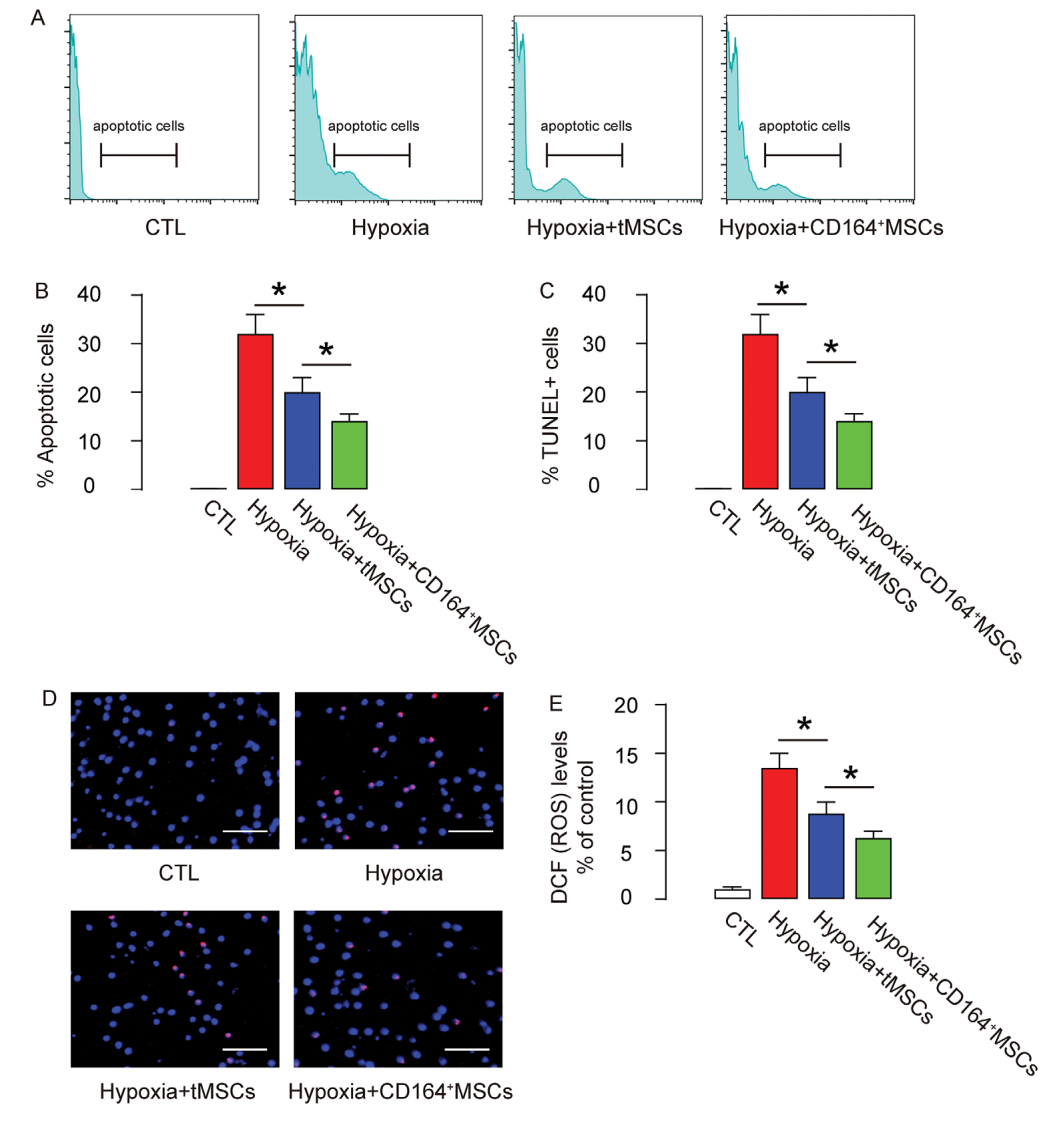
**Reduced cell apoptosis and ROS formation by CD146+MSCs against hypoxia in cardiac muscle cells.** (**A-D**) The apoptosis of CMC in an Annexin V-apoptosis assay (**A-B**), shown by representative flow charts (A), and by quantification (B), and in a TUNEL assay (**C-D**), shown by quantification (**C**), and by representative images (**D**). (**E**) ROS levels by DCF-DA assay. *p<0.05. N=5. Scale bars are 50 µm.

Next, we analyzed the apoptosis of CMC in an Annexin V-apoptosis assay and in a TUNEL assay. We found that hypoxia induced CMC apoptosis, which was attenuated by co-culture with tMSCs but further significantly attenuated by co-culture with CD164+MSCs, in an Annexin V-apoptosis assay (Figure 5A-B) and in a TUNEL assay (Figure 5C-D). ROS levels have been used as a signature for cell aging. Here, we found that the increased ROS levels by hypoxia were significantly attenuated by co-culture with tMSCs but further significantly attenuated by co-culture with CD164+MSCs (Figure 5E). Together, these data suggest that CD146+MSCs may have better protective effects on CMCs against hypoxia through rejuvenating CMCs.

## DISCUSSION

MSCs have potential in promoting tissue repair and regeneration in adulthood, largely resulting from their multipotent and stem cell properties potentials [22]. The use of MSCs in recovering or regenerating injured hearts have some successes in pre-clinical researches, but the relatively big difference in the therapeutic outcome among various studies prevents its translation into clinical trials. Of note, this potent variation may result from the use of different preparation of MSCs in different studies, taking into account that MSCs are not a group of identical cells while different MSC subtypes may be prone to different cell lineage differentiation in future [19].

In the current study, we hypothesize that MSCs may have a subpopulation that expresses CD146, and this subpopulation (CD146+MSCs) may be better for protecting and regenerating injured hearts than tMSCs. This hypothesis stems from 2 supporting evidence. First, CD146 in a marker that expresses in capillary pericytes, and pericytes have been shown to produce significant levels of MMPs or associated proteins that control the degradation of extracellular matrix to suppress fibrosis [20]. Second, pericytes are known to play a positive role in angiogenesis [23], which may be also important for post-MI regeneration of CMCs [21]. Our data support our hypothesis, and clearly showed that CD146+ subpopulation of MSCs has a more pronounced beneficial effect on heart function after MI, consistent with previous results showing neural ganglioside GD2+ MSCs being a subpopulation with feature of primitive precursor cells [24,25], CD133+/ATP-binding cassette sub-family G member 2+/C-X-C chemokine receptor type 4+ MSCs being potent neurogenic and neuro-protective for traumatic brain injury [26], CD106+ MSCs with potential immunomodulatory properties [27]. Hence, our study demonstrates that use of MSC subpopulation may improve the therapeutic outcome in certain situations.

CMCs are generated from born and a potential stem/progenitor cell for CMC does not exist. Aging of CMCs is associated with a decline in cell proliferation, an increase in ROS formation and apoptosis. Some previous studies have highlighted rejuvenation of CMCs may play a critical role in MSC-mediated protection and recovery of the injured heart [9–11]. For example, Li et al. has shown that Sca-1+ bone marrow cells from young mice home better to the infarcted heart than those from old mice and these cells improved CMC function through activation of PDGFR-beta/Akt signaling pathway [10]. Moreover, this group showed that the rejuvenation of the aged heart by Sca-1+ bone marrow cells may be required an epithelial-mesenchymal transition of the CMCs [11]. Here, our data showed that CD146+MSCs have more pronounced effects on CMCs in promoting proliferation and suppressing apoptosis, both *in vivo* in MI model and *in vitro* under hypoxia condition. Moreover, ROS levels in CMCs under hypoxia were improved in a higher degree by CD146+MSCs. These data are consistent with a rejuvenating state of CMCs by CD146+MSCs.

To the best of our knowledge, this study is the first one to reveal CD146+ population of MSCs has a better therapeutic effect on MI and the results here may be examined in future to challenge its applicability in clinic.

## MATERIALS AND METHODS

### Protocol approval

All the cell and animal experimental methods have received approval from the research committee at the Shanghai Chest Hospital.

### Manipulation of mouse MSCs

MSCs were obtained from euthanized male C57/BL6 mice of 10 weeks of age (Shanghai Laboratory Animal Center, Shanghai, China) via flushing the bone cavity with PBS using a 23-gauge springe. After centrifugation, cells were cultured in DMEM low glucose (Invitrogen, Beijing, China), 10% FBS (Sigma-Aldrich, Shanghai, China), 100 U/ml penicillin (Invitrogen), 100g/ml streptomycin (Invitrogen), 5 ng/ml bFGF (Invitrogen) and 5 ng/ml EGF (Invitrogen). After a 5-passage removal of non-adherent cells, the adherent MSCs were obtained and characterized by analysis for surface marker expression by flow cytometry. Differentiation capacity of MSCs was assessed by inducing osteocyte, adipocyte and chondrocyte differentiation with corresponding kits (American Type Culture Collection (ATCC), Rockville, MD, USA; Catalog number: PCS-500-052, PCS-500-050 and PCS-500-051), and evaluated by Von kossa staining, Oil red O staining and Alcian blue staining, respectively.

### Flow cytometry

The antibodies for surface marker analysis for MSCs or isolation of CD146+MSCs are PEcy7-conjugated anti-Sca-1, CD105, CD90, CD45, CD34, HLA-DR and FITC-conjugated anti-CD146 (Becton-Dickinson Biosciences, Shanghai, China). For isolation of CMCs, PE-conjugated anti-Troponin T (cTnT; Becton-Dickinson Biosciences) was used in flow cytometry. Assessment of apoptosis was labeled with a Dead Cell Apoptosis Kit with Annexin V FITC and propidium iodide (PI) (Invitrogen; V13242), and analyzed by flow cytometry. Both pro-apoptotic cells and apoptotic cells were counted. Assessment of proliferation was done with a BrdU Staining Kit for Flow Cytometry (Thermo Fisher Scientific, Inc., Waltham, MA, USA; 8811-6600-42). Reactive oxygen species (ROS) levels were measured in live cells with a dichlorodihydrofluorescein diacetate (DCFH-DA) method that converts 2’,7’-dichlorofluorescin diacetate (DCFDA) to oxidized 2’, 7’ -dichlorofluorescein (DCF), which is detected by by flow cytometry. Flow cytometry data were analyzed and presented with FlowJo software (Flowjo LLC, Ashland, OR, USA).

### *In vitro* co-culturing

The CMCs for co-culture were isolated from 10-week-old male C57/BL6 mice (Shanghai Laboratory Animal Center). Briefly, mouse hearts were dissected out, PBS-washed, spliced into small pieces, and then incubated in a 37 °C water bath with a magnetic bar for 12 minutes, followed by digestion by fresh pre-warmed 0.25% trypsin at 37 °C for 6 minutes. The dissociated cells that pass a 66µm filter plated into the co-culturing system. Four conditions were applied. CMCs were plated at the lower plate. Condition 1, upper plate contained control media in normoxia condition (CTL). Condition 2, upper plate contained control media in hypoxia condition (Hypoxia). Condition 3, the upper plate contained same number of tMSCs (to the number of CMCs in lower plate) in hypoxia condition (Hypoxia+tMSCs). Condition 4, the upper plate contained same number of CD164+MSCs (to the number of CMCs in lower plate) in hypoxia condition (Hypoxia+CD164+MSCs). CMCs were analyzed 24 hours after culturing.

### MI in mice and transplantation of MSCs

MI was induced in male C57/BL6 mice at 10 weeks of age by ligation of the left anterior descending artery, as described [21]. One hour after ligation, mice received injection of saline (Sham or MI) or 5X10^5^ tMSCs (MI+tMSCs) or 5X10^5^ CD164+MSCs (CD164+MSCs) at 5 sites along the ligation. The mice were then kept for 4 weeks before analysis.

### Hemodynamic assessments

After anesthetization, ventricular catheterization was performed on mice. A 1.2F pressure volume catheter was connected to an Advantage PV-loop system, and then inserted into the left ventricle via right carotid artery. The hemodynamic parameters, LV pressure and positive maximal pressure derivative (+dP/dt) were measured. The end systolic pressure-volume relationship (ESPVR) was obtained after and before inferior vena cava occlusion.

### Quantitative real-time PCR (RT-qPCR)

Total RNA was extracted using the RNeasy kit (Qiagen, Shanghai, China). Complementary DNA preparation and quantitative real-time PCR (RT-qPCR) were performed using primers that were all purchased from Qiagen. Data were collected and analyzed using 2-△△Ct method. Values of genes were first normalized against GAPDH, and then compared to the experimental controls.

### ELISA

ELISA for CD146 was done using an ELISA kit for CD146 (LSBio, Seattle, WA, USA).

### TUNEL and Masson's trichrome staining

TUNEL (terminal deoxynucleotidyl transferase dUTP nick end labeling) staining was performed using a TUNEL kit (Abcam, Shanghai, China; Ab66110). Masson's trichrome staining on heart sections were performed with a Masson's trichrome staining kit (Sigma-Aldrich; HT15). The infarction level was presented as the ratio of the infarcted area to the total left ventricle area.

### Statistical analysis

All values represent the mean ± standard deviation (SD). Statistical analysis of group differences was carried out using a one-way analysis of variance (ANOVA) test followed by the Fisher’s Exact Test to compare two groups (GraphPad Software, Inc. La Jolla, CA, USA). A value of p<0.05 was considered statistically significant after Bonferroni correction.
